# Review of: *Wide Neighborhoods: A Story of the Frontier Nursing Service*

**DOI:** 10.13023/jah.0401.07

**Published:** 2022-02-13

**Authors:** Tauna Gulley

**Affiliations:** The University of Pikeville

**Keywords:** Appalachia, book review, Frontier Nursing Service

## Abstract

The Journal of Appalachian Health is committed to reviewing published media that relates to contemporary concepts affecting the health of Appalachia. Access to care remains one of the biggest challenges to Appalachian Health. The book, *Wide Neighborhoods: A Story of the Frontier Nursing Service* by Mary Breckinridge, is a well-known title that seems as relevant today as it was in 1952.

## MEDIA TYPE: BOOK

### CITATION

Breckinridge M. Wide Neighborhoods: A Story of the Frontier Nursing Service. Lexington KY: The University Press of Kentucky; 1952.

ISBN-10: 0813101492 / ISBN-13: 978-0813101491; Cost: $30.00 paperback, $22.99 Kindle edition, hardcover varies.

### ABOUT THE REVIEWER

Tauna Gulley, PhD, RN, FNP-BC, CNE, is a professor of nursing at the University of Pikeville. She has more than 20 years of experience as a nurse educator and practicing nurse practitioner in rural southwest Virginia. Most recently, she coauthored *Better for Being with You: A Philosophy of Care*, a book about the life of Sr. Bernie Kenny, founder of the Health Wagon. Dr. Gulley has provided health care to individuals residing in rural areas since 1989, beginning as a home health nurse, then as a registered nurse in the intensive care unit, and since 1998 as a family nurse practitioner.

### ABOUT THE AUTHOR

Mary Breckinridge made a significant positive impact in the American healthcare landscape as a nurse midwife and founder of the Frontier Nursing Service during 1925 in rural Kentucky. She was an innovator with a vision to improve health outcomes for families and implemented the trained nurse-midwife model to care for women and children. Ms. Breckenridge’s purpose in writing her autobiography was to share life experiences that took her across the world and to describe how those events shaped the development of the Frontier Nursing Service in Leslie County, Kentucky.

### THE REVIEW

The book is well read by many, including current and former students attending Frontier Nursing University, formerly Frontier Nursing Service. Over time, The Frontier Nursing Service evolved into a hospital and a school of nursing that continue to offer graduate studies for midwifery, psychiatric mental health nursing, and primary care nursing.

This book is for any health service professional caring for people living in rural areas. Breckinridge provides inspiration for the future while being grounded in the challenges of the past—many of which seem all too real today. While originally published in 1952, topics are relevant to the issues facing rural health and health care currently. Themes of addressing health disparities through culturally competent care, providing preventative care to prevent long-term sequelae, and developing caring systems that serve people and families as part of population health as well as unique individuals resonate throughout.

Ms. Breckinridge describes her childhood during which she frequently traveled with her family. Her grandfather, John C. Breckinridge, was Vice President of the U.S., and her father held political office. This allowed her and her family to travel to several countries and enjoy various experiences.

Prior to arriving in Kentucky, Ms. Breckinridge traveled to Scotland to learn about rural nursing. It was here that she visited the “blackest home she ever saw” where a dirt floor and chimney soot contributed to the blackness. She was surprised by the level of poverty one could live in. Ms. Breckinridge described having tea with the lady of the home and how special she made her feel as a guest.

Uncommon for her time, Breckenridge was a nurse, businesswoman, and community activist. She worked tirelessly to finance her home, Wendover, which became a refuge to many and served as the headquarters for the Frontier Nursing Service. She believed that when we take care of women and children, we promote health and prosperity in our communities; this work was unprecedented in her time. She also collected and distributed food and clothes via horseback. She founded the first hospital in Hyden, Kentucky, in 1928 and the hospital is open today.

Breckenridge’s writing evokes vivid images of her professional life and evidences her lifelong love of literature. The book also includes black-and-white photos displaying images of nurses caring for children in private homes and caring for individuals in the Mary Breckenridge Hospital.

#### Relevance to Health in Appalachia

Much like today, access to health care in rural areas was challenging when Mary Breckinridge founded the Frontier Nursing Service in 1925. Her hope was that the Frontier Nursing Service would provide care for children and families in remote areas. Their efforts decreased mortality rates for women and children. Ms. Breckinridge was a believer in keeping records, reviewing data, and studying outcomes. These lessons and outcomes—that are considered phenomenal by modern standards—continue to serve as a blueprint for what is possible. Use of her ideas in the present are still relevant as we aim to improve healthcare access and outcomes in rural Appalachia.

